# Marine Cryptophytes Are Great Sources of EPA and DHA

**DOI:** 10.3390/md16010003

**Published:** 2017-12-26

**Authors:** Elina Peltomaa, Matthew D. Johnson, Sami J. Taipale

**Affiliations:** 1 Department of Environmental Sciences, University of Helsinki, Niemenkatu 73, 15140 Lahti, Finland; 2 Biology Department, Woods Hole Oceanographic Institute, 266 Woods Hole Road, Woods Hole, MA 02543, USA; mattjohnson@whoi.edu; 3 Department of Biological and Environmental Science, University of Jyväskylä, P.O. Box 35 (YA), 40014 Jyväskylä, Finland; sami.taipale@jyu.fi

**Keywords:** microalgae, polyunsaturated fatty acids, omega-3, omega-6, sterols, functional foods, nutraceuticals

## Abstract

Microalgae have the ability to synthetize many compounds, some of which have been recognized as a source of functional ingredients for nutraceuticals with positive health effects. One well-known example is the long-chain polyunsaturated fatty acids (PUFAs), which are essential for human nutrition. Eicosapentaenoic acid (EPA) and docosahexaenoic acid (DHA) are the two most important long-chain omega-3 (ω-3) PUFAs involved in human physiology, and both industries are almost exclusively based on microalgae. In addition, algae produce phytosterols that reduce serum cholesterol. Here we determined the growth rates, biomass yields, PUFA and sterol content, and daily gain of eight strains of marine cryptophytes. The maximal growth rates of the cryptophytes varied between 0.34–0.70 divisions day^−1^, which is relatively good in relation to previously screened algal taxa. The studied cryptophytes were extremely rich in ω-3 PUFAs, especially in EPA and DHA (range 5.8–12.5 and 0.8–6.1 µg mg dry weight^−1^, respectively), but their sterol concentrations were low. Among the studied strains, *Storeatula major* was superior in PUFA production, and it also produces all PUFAs, i.e., α-linolenic acid (ALA), stearidonic acid (SDA), EPA, and DHA, which is rare in phytoplankton in general. We conclude that marine cryptophytes are a good alternative for the ecologically sustainable and profitable production of health-promoting lipids.

## 1. Introduction

Consumers’ growing awareness of healthy products has promoted the development of novel natural sources of functional ingredients. Microalgae have been suggested to be among the most promising sources, since they can be used to produce many kinds of biomolecules with high nutraceutical value [1,2,3]. Microalgae can be grown in controlled conditions, allowing the production of biomass with a constant biochemical composition and eliminating the risk of chemical contamination of the biomass. Thus, the biotechnology of microalgae has gained considerable importance in recent decades.

To date, many commercial nutraceuticals, dietary supplements, and functional ingredients as well as pigments are produced using green algae, e.g., *Chlorella*, and cyanobacteria, e.g., *Spirulina*. These taxa are fast growing and rich in, for example, amino acids, but they are low in some of the most desirable biomolecules such as long-chain polyunsaturated omega-3 fatty acids (ω-3 PUFAs), which are a specific group of polyunsaturated fatty acids in which the first double bond is located between the third and fourth carbon atom counting from the methyl end of the fatty acid. Only plants can synthesize PUFAs, which means that consumers must obtain them from their diet [4].

The four types of ω-3 PUFAs mostly involved in human physiology are α-linolenic acid (ALA; 18:3:ω3), stearidonic acid (SDA, 18:4:ω3), eicosapentaenoic acid (EPA; 20:5:ω3), and docosahexaenoic acid (DHA; 22:6:ω3). The long-chain EPA and DHA can be obtained only from certain organisms, mainly of aquatic origin, whereas all terrestrial plants produce and contain fairly high amounts of ALA, which is the precursor to the ω-3 series fatty acids (FAs) [5]. The human body cannot produce short-chain ω-3 on its own, and converts the short-chain ω-3 to the long-chain versions, especially DHA, inefficiently (i.e., only 3–4% is converted) [4]. Thus, EPA and DHA are often determined as essential dietary nutrients. They are both needed for normal metabolism, and they have been implicated in the reduction of cardiovascular diseases like arrhythmia, stroke, and high blood pressure, and can ameliorate renal diseases, depression, dementia, rheumatoid arthritis, and asthma, and are essential for normal fetal brain development as well as growth and development of infants/children [1,6].

The ω-6 PUFAs have been shown to promote health in some cases, e.g., reduce nerve pain of diabetic patients and help boys with symptoms of attention deficit hyperactivity disorder (ADHD) [7,8]; however, excess dietary gain of ω-6 PUFAs is known to promote cardiovascular disease, cancer, and inflammatory and autoimmune diseases in humans [9]. In the Western diet, ω-6 PUFA consumption has become progressively much higher than that of ω-3 PUFA due to increased consumption of vegetable oils rich in linoleic acid (LA; 18:2ω6; precursor of the long chain ω-6 FAs) [10]. The optimal dietary ω-6/ω-3 ratio should be around 1-4:1, but in the Western diet it varies between 10:1 and 20:1 [11,12]. This is significant, since both ω-6 and ω-3 elongation need the same enzymes and, thus, in humans, the excess consumption of ω-6 FAs reduces the already low conversion rate of short-chain ω-3 ALA to long-chain EPA and DHA by 40–50% [13].

So far, fish oil has been the main commercial source of EPA and DHA, but alternative sources are needed since the global consumer needs cannot be satisfied by the current global fish stock harvests, and also the practice of using fish as feed at fish farms is nonsustainable. Furthermore, fish oil is not suitable for vegetarians or people allergic to fish, it has an odor unpleasant to some people, and it may contain lipid-soluble environmental pollutants. Fish oil quality also varies depending on the fish species, season, and geographical location [14]. Like humans, fish obtain EPA and DHA from their diet, basically from microalgae, some of which could be used in commercial PUFA production.

Besides PUFAs, many algae are also rich in other lipids, such as phytosterols. Phytosterols have chemical structures similar to cholesterol, but they are effective in reducing serum cholesterol levels, and they may also prevent Alzheimer’s disease, suppress the growth of colonic tumors, and have beneficial effects on prostate disorders [15,16]. Stigmasterol, campesterol, and sitosterol are the main molecular species of phytosterols, and stigmasterol is claimed to be the most valuable of these three due to its health-promoting benefits [17]. Phytosterols can be found both in terrestrial and aquatic plants and algae. Similar to ω-3 PUFAs, dietary consumption is the only source of phytosterols found in plasma of humans [18].

Even though microalgae are potentially very promising sources of lipids, the present challenge is to find an economically viable way to use them for lipid production, and searching for the most suitable species/strains plays an important role in this. Currently, *Nannochloropsis* (eustigmatophyte) species are suggested to be among the most promising EPA and DHA producers for commercial applications [2,19]. However, many microalgae are known to be able to accumulate lipids in high amounts, and high EPA and DHA concentrations have been detected in both marine and fresh waters in cryptophytes, dinoflagellates, and diatoms [20]. These three groups represent distinct phylogenetic branches in the eukaryotic tree of life relative to green algae [21,22], and, thus, likely produce unique cocktails of nutraceuticals.

Due to their high PUFA, sterol, and amino acid concentrations, the nutritional value of cryptophytes has been proven to be of utmost importance in aquatic food webs [1,23,24,25]. This suggests that cryptophyte algae could offer a potential source of lipids also for nutraceutical purposes. There are already several publications reporting high PUFA concentrations in cryptophytes; however, most of these studies do not report the total amounts of FAs per biomass but only PUFA percentages of the total FAs [1,26,27,28]. Similarly, most studies investigating FA content and composition in microalgae do not report growth rates. This may give the wrong impression of their potential in profitable PUFA production. Most cryptophytes are known to grow fairly slowly (growth rates well below 0.8 div. day^−1^) [27,29,30], which may hinder their usability in commercial production. However, in suitable conditions some strains are reported to have higher growth rates (e.g., 1.2 div. day^−1^) [31]. The cell size of most cryptophyte species is small (below 500 µm^3^), and they do not possess heavy cell wall structures, which means that the cell biomass is low compared with that of many diatoms and dinoflagellates. However, due to the lack of recalcitrant cell wall, cryptophytes are easier to break and process further for commercial purposes than diatoms, dinoflagellates, or, for example, *Nannochloropsis* [32]. Since the cultivation of marine algae has the benefits of using non-potable water and non-arable land, we chose to focus on marine cryptophytes in this study. We analyzed the PUFA and sterol contents of eight marine cryptophyte strains and estimate their usability (e.g., growth rate and biomass production) for commercial lipid production.

## 2. Results and Discussion

### 2.1. Growth and Biomass Production

As expected, all species showed moderate growth rates between 0.34–0.70 div. day^−1^ in the exponential growth phase (Table 1). However, the growth rates were very well comparable with the ones detected for *Nannochloropsis* species, (0.04–0.60 div. day^−1^) [19]. Similarly, the observed growth rates were competitive with strains of polar and temperate microalgae, including the prymnesiophyte *Emiliania huxleyi*, prasinophyte *Pyramimonas* sp., and the raphidophyte *Fibrocapsa japonica*. The diatoms *Chaetoceros brevis* and *Thalassiosira weissflogii* have been suggested for EPA and DHA production by Bolen et al. [33] and also have lower growth rates (range 0.14–0.49 div. day^−1^), as do tropical *Nannochloropsis* sp., *Isochrysis* sp., and *Tetraselmis* sp., which have been suggested for scale-up production by Huerlimann et al. [34] (range 0.10–0.41 div. day^−1^). The daily volumetric biomass yields of the cryptophyte strains differed remarkably, being highest in *Chroomonas mesostigmatica* and *Storeatula major* (3.40 and 3.23 mg dry weight (DW) L^−1^ day^−1^) and lowest in *Hemiselmis* sp. (0.28 mg DW L^−1^ day^−1^; Table 1). In terms of biomass production, the studied cryptophytes were not competitive with *Nannochloropsis* species or commercially grown green algae, which can reach 10–100 times higher volumetric yields [35,36]. However, it needs to be kept in mind that, unlike *Nannochloropsis* and many other algae, cryptophytes do not have strong and heavy cell wall structures, which would also increase their cellular biomass. Thus, the comparisons of biomass yields between different algal classes can be rather misleading. Furthermore, we did not try to optimize the growth rates or biomass production of cryptophytes in this study, and thus cannot fully conclude the inferiority of cryptophytes in terms of biomass production.

### 2.2. Polyunsaturated Fatty Acids

The proportion of ω-3 PUFAs (73.9 ± 7.0% of all FAs) was high in all of the studied strains, which is in accordance with previous studies on cryptophytes and very competitive compared with microalgae in general [26,27,28,37]. The highest ω-3 PUFA proportions were found in *S. major* (81.1 ± 0.3% of all FA) and *Proteomonas sulcata* (80.3 ± 1.9% of all FA). These two strains together with *Guillardia theta* and *Hemiselmis* sp. also had the highest amount of ω-3 PUFAs in proportion to dry weight (4.9 ± 0.6% of DW; Table 2).

The contribution of the short-chain ALA (average 51.1 ± 7.0% of all FAs) and SDA (average 21.0 ± 5.8% of all FAs) varied a little between species, but did not differ statistically between them. However, the proportion of long-chain EPA (average 19.1 ± 4.6% of all FAs) varied significantly between the strains, as also did DHA (average 8.8 ± 4.9% of all FAs; Table 2). The studied marine species had higher EPA and DHA proportions than has been previously found in the brackish cryptophyte *Rhodomonas baltica* by Patil et al. [35] (EPA 13.1% and DHA 0.6% of all FAs). In comparison to freshwater species, marine cryptophytes had equal amount of EPA, but more DHA than freshwater strains [24,25]. The combined percentage of EPA and DHA is important for commercial purposes. Together the EPA and DHA of the studied marine cryptophytes represented 20.8 ± 6.7% of all FAs, which is competitive to the already commercially employed algae or species suggested as suitable for commercial EPA and DHA production (EPA + DHA range 5–57% of total FAs) [2,3]. Furthermore, the contribution of EPA and DHA was similar to the reported values of *Nannochloropsis* sp. (26.7%) that has so far been considered to be one of most promising strains for commercial EPA and DHA production [2]. The advantage of marine cryptophytes is that they contain a high proportion of both EPA and DHA, whereas *Nannochloropsis* species contain mainly EPA [34].

The studies dealing with algal FA production often report results as proportions of PUFAs or total FAs as we also did above. This makes it easy to compare between the strains, but does not actually tell anything about the true gain of the FAs. Thus, we calculated the synthesized amount of FAs and per volume per day (µg L^−1^ day^−1^). When calculated this way, *S. major* and *C. mesostigmatica* were the best producers of ALA (45 ± 1.1 µg L^−1^ day^−1^; Table 3), even though the highest ALA content (2.6% of DW) was found in *Hemiselmis* sp. Both *S. major* and *C. mesostigmatica* produced up to 10 times more ALA daily than any other strain. Similarly, the SDA content was highest in the *S. major* (2.4 ± 0.01% of DW) followed by *C. mesostigmatica* and *G. theta*. When taking into account the time and volume, *S. major* and *C. mesostigmatica* were also the two most efficient producers of SDA (76 ± 3.3 µg L^−1^ day^−1^; Table 3), whereas the SDA gain was lowest in *G. theta*. Due to the four double bond structure of SDA, it does not require the rate-limiting enzyme desaturase to convert to EPA in humans, which means that dietary SDA is a better precursor of EPA than ALA [38,39]. Thus, in addition to high EPA, the high SDA content even further increases the value of certain cryptophytes as potential commercial sources of ω-3 PUFAs. However, purely as SDA producers, algae compete with, e.g., soy that can be used for enriched SDA oil production [39].

The content of EPA was highest (1.1 ± 0.2% of DW) in *Hemiselmis* sp. and *C. mesostigmatica*, which is more than previously found in *R. baltica* or *Oocystis* sp. (0.11–0.4% of DW) [35], but less than found from freshwater cryptophytes [24,25]. In relation to gain, *S. major* and *C. mesostigmatica* were the two most efficient EPA synthesizers (29 ± 3.6 µg L^−1^ day^−1^), and their EPA gain was double compared with those of the second-best strains, i.e., *Teleaulax amphioxeia* and *Rhodomonas salina* (17 ± 0.9 µg L^−1^ day^−1^). The DHA content was highest (0.5 ± 0.08% DHA of DW) in *P. sulcata*, *S. major* and *T. amphioxeia*. Their results were similar to previous measurements of freshwater strains, but higher than found in brackish strains [25,35]. However, when calculated as daily gain, *C. mesostigmatica* was shown to be a poor DHA producer (2.7 ± 0.2 µg L^−1^ day^−1^), whereas *S. major* was able to synthesize high amounts of DHA (17 ± 0.2 µg L^−1^ day^−1^). Also, *P. sulcata*, *R. salina* and *T. amphioxeia* were better DHA producers (9.7 ± 0.8 µg L^−1^ day^−1^) than *C. mesostigmatica*. Thus, among all studied strains, *S. major* seems to be the superior species for ALA, SDA, EPA, and DHA production.

In commercially available food supplements, the EPA/DHA ratios are around 1.5 [40]. However, the optimal EPA/DHA ratios have not been rigorously examined, and, thus, it is not known if this is the most desirable ratio. Most of the studied cryptophytes had a similar or slightly higher EPA/DHA ratios compared to established commercial products (Table 2), while the highest was observed in *C. mesostigmatica* (EPA/DHA = 11.6) and lowest in *R. salina* (EPA/DHA 0.5). 

The proportions of ω-6 PUFAs of all FAs varied greatly between strains, and was highest (19.8 ± 0.1% of all FAs) in *R. salina* and lowest (0.7 ± 0.01% of all FAs) in *Teleaulax acuta* (Table 4). The ω-6 PUFA content (% of DW) was generally low in all strains (0.02–1.0% of DW), but differed significantly among strains. Total ω-6 PUFAs were highest in *R. salina* (1.0 ± 0.04% of DW), but were also high in *C. mesostigmatica* (0.53 ± 0.01% of DW) and *G. theta* (0.56 ± 0.06% of DW) (Table 4). The total ω-6 content of *R. salina* was higher than previously reported for freshwater and brackish cryptophytes [25,35]. Usually, such high amounts of ω-6 are found in brown algae [41]. More specifically, the highest contents of LA (0.6 ± 0.02% of DW) and arachidonic acid (ARA; 20:4ω6, 0.16 ± 0.01% of DW) were found in *R. salina*. Docosapentaenoic acid (DPA; 22:5ω6) concentrations were, in general, very low, and highest in *R. salina* (0.03 ± 0.01% of DW) and *P. sulcata* (0.04 ± 0.01% of DW, Table 4). When calculated as daily gain, the highest total ω-6 PUFA was attained in *S. major* (43 µg L^−1^ day^−1^; Table 3).

Modern western diets are deficient in ω-3 PUFAs, but have excessive amounts of ω-6. Thus, supplemental products are needed for amending the high ω-6/ω-3 ratios. In the studied cryptophytes, the lowest ω-6/ω-3 ratio was found in *T. acuta* (1:126) and the highest in *R. salina* (1:3; Table 4). These values are well below the suggested dietary ω-6/ω-3 ratio (1-4:1) [11], and substantially lower than reported for some brown algae (up to 3.8:1) [41]. Thus, despite the surprisingly high ω-6 PUFA concentrations in some of studied cryptophytes, all of them are suitable for commercial PUFA production and especially for products that are designed for balancing the dietary ω-6/ω-3 ratios.

### 2.3. Phytosterols

Three different phytosterols (crinosterol, brassicasterol, and stigmasterol) were found in the studied cryptophytes (Table 5). Crinosterol and brassicasterol are known to act as anti-ageing factors [42], whereas stigmasterol is reported to be most valuable of the phytosterols and have anti-inflammatory effects, preventing, for example, osteoarthritis [15,17]. In general, the total sterol concentrations were low in the studied cryptophytes (0.09 ± 0.04% of DW), and only *C. mesostigmatica* contained all of the three observed sterols (Table 5). Thus, the daily gains were low (range 0.37–4.36 µg L^−1^ day^−1^; Table 3). The obtained results are in line with the ones reported for diatoms (total sterols 0.06–0.57% of DW) [43], but significantly lower than reported for seaweeds (0.6–2.3% of DW) [44]. This suggests that the studied cryptophytes—or perhaps algae in general—are not suitable for profitable sterol production. However, due to the general benefits of phytosterols in human health, even low sterol contents may improve their value as ingredients for functional foods or nutraceuticals [15,16].

## 3. Materials and Methods

### 3.1. Growth Rate and Biomass Production

The eight marine cryptophyte strains (Table 1) were grown in F/2 medium in 400 mL plastic tissue culture flasks. Each strain had two replicates, which were kept at 15 °C and under a 14 h:10 h light/dark cycle at a light level of 50 µmol quanta s^−1^ m^−2^. The growth rates were calculated for the exponential growth phase using three sampling points and Equation (1). Biomass production (as mg dry weight L^−1^ day^−1^) of the cultures was determined from freeze-dried samples taken at the time of lipid sampling near the end of the exponential growth phase. 

µ = ln(cells_Tx_/cells_T0_) (1)

### 3.2. Lipid Extraction

The samples for lipid analyses were collected by centrifugation (3200 rpm for 10 min) near the end of the exponential growth phase. The obtained pellets were placed into −80 °C until freeze-drying. The lipids were extracted from the freeze-dried samples no longer than three weeks after the sampling. Two replicates of homogenized biomass samples (1–3 mg) were extracted using chloroform/methanol 2:1 (by vol). Samples were sonicated for 10 min to maximize extraction results, after which samples were vortexed and centrifuged for 5 min at 2000 rpm. The liquid part was divided into two equal parts, one of which was used for fatty acid analysis and the other for sterol analysis.

### 3.3. Fatty Acid Transesterification and Analysis by GC-MS

Toluene and 1% sulfuric acid in methanol were used for the transesterification of fatty acid methyl esters (FAME). The samples were heated at 90 °C for 90 min, after which 1.5 mL of 2% of KHCO_3_ and 2 mL hexane was added. The tubes were vortexed and centrifuged (2 min at 1500 rpm) and the upper layer was collected for analysis. FAMEs were analyzed with a gas chromatograph (Shimadzu Ultra, Kyoto, Japan) equipped with mass detector (GC-MS), using helium as a carrier gas and an Agilent^®^ (Folsom, CA, USA) DB-23 column (30 m × 0.25 mm × 0.15 µm). Fatty acids were identified by the retention times (RT) and using specific ions [24]. All of the detected fatty acids and their concentrations (as in µg FA in mg DW) in the studied cryptophyte strains are shown in Supplementary Table S1.

### 3.4. Silylation of Sterols and Analysis by GC-MS

The extracted lipids were diluted in 100 µL of pyridine and silylated with 70 µL of *N*,*O*-bis[trimethylsilyl trifluoroacetamide] (BSTFA), trimethylchlorosilane (TMCS) at 70 °C. Trimethylsilyl (TMS) derivatives of sterols were analyzed with GC-MS (Shimadzu) equipped with A Phenomenex (Torrance, CA, USA) ZB-5 Guardian column (30 m × 0.25 mm × 0.25 μm). Sterols were identified using characteristic ions [45].

### 3.5. Statistics

The differences between the growth rates and lipid production between the cryptophyte strains were studied with ANOVA and Tukey’s honestly significant difference (HSD) post hoc test. Levene’s test was used for testing the homogeneity of variances. All the statistical analyses were performed using R (stats packages: AOV, and TukeyHSD functions) [46] or IBM SPSS Statistics 22 for Windows (IBM Corp., Armonk, NY, USA).

## 4. Conclusions

The increasing interest in exploiting microalgae as a source of commercial products with positive health effects has led to an ongoing search for high production potential of, e.g., essential lipids. In this study we focused on ω-3 and ω-6 PUFAs as well as sterol production by eight marine cryptophytes. Cryptophytes do not have a recalcitrant cell wall, and, thus, they are easier to break and process further for commercial purposes than many of the already commercially employed algae. In addition, the cultivation of marine species for nutritional purposes can utilize non-arable lands and water resources considered unsuitable for agriculture or direct human consumption. We conclude that the marine cryptophytes offer a great source of EPA, DHA, and other ω-3 PUFAs, and that, in comparison to freshwater species, marine cryptophytes have more DHA. We found that cryptophytes also produce ω-6 PUFAs, but their proportion is low compared to that of ω-3 PUFAs, which is beneficial especially for dietary products targeted to lower ω-6/ω-3 ratios. When considering both the biomass yield and the yield of the lipids, *C. mesostigmatica* was the most promising strain for EPA production, whereas *S. major* seems promising for both EPA and DHA production. These two strains also had the highest sterol gains. However, the purpose of this study was not to optimize growth conditions, and, thus, the other studied strains could likely show even higher growth rates and biomass and lipid yields under optimized conditions.

## Figures and Tables

**Table 1:** The studied cryptophyte strains, their codes in culture collection, growth rates (divisions day^−1^) and volumetric biomass yields (mg dry weight L^−1^ day^−1^). Different letters (a–f) denote significant differences (ANOVA *p* < 0.05) between strains.

Species	Code in Culture Collection	Max. Growth Rate *	Dry Weight (mg L^−1^ Day^−1^) °
*Chroomonas mesostigmatica*	CCMP/NCMA 1168	0.65 (0.03) ^abc^	3.40
*Guillardia theta*	CCMP/NCMA 2712	0.66 (0.04) ^a^	0.56
*Hemiselmis* sp.	NRC5	0.70 (0.01) ^abcd^	0.28
*Proteomonas sulcata*	CCMP/NCMA 704	0.65 (0.01) ^abcd^	1.47
*Rhodomonas salina*	CCMP/NCMA 757	0.51 (0.01) ^bcde^	2.79
*Storeatula major*	SM or G	0.48 (0.03) ^bcde^	3.23
*Teleaulax acuta*	SCCAP K-1486	0.34 (0.01) ^f^	1.05
*Teleaulax amphioxeia*	GCEP01	0.55 (0.03) ^ab^	2.05

* Detected after 4 days for *C. mesostigmatica*, 5 days for *T. amphioxeia*, *Hemiselmis* sp. and *R. salina*, and 7 days for *G. theta*, *T. acuta*, *P. sulcata* and *S. major*. ° Detected after 5 days for *C. mesostigmatica*, 7 days for *T. amphioxeia*, *S. major* and *R. salina*, and after 9 days for *G. theta*, *T. acuta*, *P. sulcata* and *Hemiselmis* sp.

**Table 2:** The ω-3 fatty acid (FA) contents in mg dry weight (DW) (µg FA in mg DW) and proportions (%) as well as the EPA/DHA ratios of the studied cryptophytes and some other algae suggested for commercial lipid production (values derived from literature). ALA = α-linolenic acid (18:3:ω3), SDA = stearidonic acid (18:4:ω3), EPA = eicosapentaenoic acid (20:5:ω3), DHA = docosahexaenoic acid (22:6:ω3). Different letters (a–g) denote significant differences (ANOVA *p* < 0.05) between strains. The culture collection codes of the cryptophyte strains can be found in Table 1. Standard deviations for these FAs are shown in Supplemental Table S1.

Species	Total ω-3 µg FA in Mg DW	ω-3% of All FAs	ALA µg FA in Mg DW	ALA% of ω-3 FAs	SDA µg FA in Mg DW	SDA% of ω-3 FAs	EPA µg FA in Mg DW	EPA% of ω-3 FAs	DHA µg FA in Mg DW	DHA% of ω-3 FAs	EPA/DHA Ratio	EPA + DHA% of All FA
*Chroomonas mesostigmatica*	45.5 ^abcd^	71.1 ^c^	13.5 ^a^	60.3	21.7 ^a^	17.4	9.3 ^a^	20.5 ^d^	0.8 ^b^	1.7 ^g^	11.6 ^a^	15.8 ^c^
*Guillardia theta*	47.8 ^ac^	65.4 ^b^	19.7 ^a^	56.7	19.5 ^ab^	25.4	7.1 ^ab^	14.9 ^a^	1.4 ^b^	3.0 ^e^	5.1 ^b^	11.7 ^d^
*Hemiselmis* sp.	58.8 ^†^	70.1 ^†^	26.2 ^†^	53.2	16.8 ^†^	20.5	12.5 ^†^	21.2 ^f^	3.0 ^†^	5.1 ^†^	4.2 ^†^	18.5 ^b^
*Proteomonas sulcata*	48.2 ^bc^	80.3 ^a^	16.4 ^a^	58.5	19.4 ^ab^	16.2	6.1 ^ab^	12.7 ^e^	6.1 ^a^	12.6 ^b^	1 ^c^	20.4 ^b^
*Rhodomonas salina*	33.7 ^abd^	64.2 ^b^	8.6 ^b^	48.8	15.4 ^b^	22.8	5.8 ^b^	17.2 ^g^	3.8 ^c^	11.2 ^c^	0.5 ^d^	18.2 ^bc^
*Storeatula major*	51.4 ^abc^	81.1 ^a^	21.5 ^d^	41.9	24.3 ^a^	32.1	8.2 ^ab^	16.0 ^g^	5.2 ^ac^	10.0 ^d^	1.6 ^c^	21.1 ^b^
*Teleaulax acuta*	25.3 ^d^	79.6 ^a^	3.7 ^c^	46.2	11.3 ^c^	13.4	6.6 ^ab^	26.0 ^c^	3.6 ^c^	14.3 ^a^	1.8 ^c^	32.1 ^a^
*Teleaulax amphioxeia*	36.0 ^abcd^	79.6 ^a^	7.0 ^b^	43.3	15.8 ^b^	20.5	8.5 ^ab^	23.6 ^b^	4.6 ^ac^	12.7 ^b^	1.8 ^c^	28.9 ^a^
*Nannochloropsis* spp. (eustigmatophyte) *		7.7–30.8 [34]						23.4–28 [2]			<0.06 [34]	26.7 [2]
*Nitzschia* spp. (diatom) *		3–9.2 [26]										
*Skeletonema* spp. (diatom) *		16.3–17.6 [26]										
*Isochrysis* spp. (prymnesiophyte) *		30.4–48.1 [26,34]										
*Tetraselmis* sp. (chlorophyte) *		8.4–31.3 [34]										
Dunaliella salina (chlorophyte) *								21.4 [2]				
Chroothece richteriana (rhodophyte) *		29.4 [37]										15.9 [37]

^†^ Could not be statistically tested. * Values from literature.

**Table 3:** The daily gain of ω-3 and ω-6 fatty acids and sterols (µg L^−1^ day^−1^) when produced with the studied cryptophytes. ALA = α-linolenic acid (18:3:ω3), SDA = stearidonic acid (18:4:ω3), EPA = eicosapentaenoic acid (20:5:ω3), DHA = docosahexaenoic acid (22:6:ω3). LA = linoleic acid (18:2ω6), ARA = arachidonic acid (20:4ω6), DPA = docosapentaenoic acid (22:5ω6). Different letters (a–d) denote significant differences (ANOVA *p* < 0.05) between strains. The culture collection codes of the cryptophyte strains can be found in Table 1.

Species	ALA µg L^−1^ Day^−1^	SDA µg L^−1^ Day^−1^	EPA µg L^−1^ Day^−1^	DHA µg L^−1^ Day^−1^	ω-3 µg L^−1^ Day^−1^	LA µg L^−1^ Day^−1^	ARA µg L^−1^ Day^−1^	DPA µg L^−1^ Day^−1^	ω-6 µg L^−1^ Day^−1^	Sterols µg L^−1^ Day^−1^
*Chroomonas mesostigmatica*	45.93 ^a^	73.83 ^a^	31.64 ^a^	2.72 ^c^	154.81 ^a^	7.83 ^b^	0.34 ^b^	0.34 ^b^	28.92 ^b^	4.36 ^a^
*Guillardia theta*	11.00 ^b^	10.89 ^c^	3.96 ^e^	0.78 ^d^	26.69 ^c^	1.84 ^c^	-	1.17 ^a^	3.13 ^c^	0.37 ^c^
*Hemiselmis* sp.	7.28 ^†^	4.66 ^†^	3.47 ^†^	0.83 ^†^	16.34 ^†^	0.72 ^†^	-	-	1.14 ^†^	0.43 ^†^
*Proteomonas sulcata*	24.08 ^c^	28.49 ^b^	8.96 ^c^	8.96 ^b^	70.77 ^b^	1.03 ^cd^	0.44 ^b^	0.59 ^ab^	2.50 ^c^	1.04 ^bc^
*Rhodomonas salina*	23.95 ^c^	42.89 ^b^	16.16 ^b^	10.58 ^b^	93.87 ^b^	15.32 ^a^	4.46 ^a^	0.84 ^a^	26.74 ^b^	2.73 ^b^
*Storeatula major*	44.26 ^a^	78.50 ^a^	26.49 ^ab^	16.8 ^a^	166.04 ^a^	5.49 ^b^	0.65 ^b^	-	43.29 ^a^	3.10 ^ab^
*Teleaulax acuta*	3.89 ^d^	11.87 ^c^	6.93 ^d^	3.78 ^c^	26.57 ^c^	0.11 ^d^	-	-	0.21 ^d^	0.37 ^c^
*Teleaulax amphioxeia*	14.35 ^bc^	32.39 ^b^	17.43 ^b^	9.43 ^b^	73.8 ^b^	1.85 ^c^	-	-	2.05 ^c^	0.92 ^c^

^†^ Could not be statistically tested.

**Table 4:** The ω-6 fatty acid contents (µg FA in mg dry weight) and proportions (%) of the studied cryptophytes as well as their ω-6:3-ratios. LA = linoleic acid (18:2ω6), ARA = arachidonic acid (20:4ω6), DPA = docosapentaenoic acid (22:5ω6). Different letters (a–f) denote significant differences (ANOVA *p* < 0.05) between strains. The culture collection codes of the cryptophyte strains can be found in Table 1. Standard deviations for these FAs are shown in Supplemental Table S1.

Species	Total ω-6 µg FA in mg DW	ω-6% of All FA	ω-6% of DW	ω-6/ω-3 Ratio	LA µg FA in mg DW	LA% of ω-6 FAs	ARA µg FA in mg DW	ARA% of ω-6 FAs	DPA µg FA in mg DW	DPA% of ω-6 FAs
*Chroomonas mesostigmatica*	8.5 ^d^	13.3 ^b^	0.9 ^b^	1:5 ^e^	2.3 ^b^	26.8 ^g^	0.06 ^a^	0.7 ^f^	0.1 ^d^	70.4 ^a^
*Guillardia theta*	5.6 ^b^	7.7 ^c^	0.6 ^c^	1:9 ^d^	3.3 ^c^	58.4 ^c^	0.03 ^c^	0.5 ^f^	2.1 ^a^	36.8 ^b^
*Hemiselmis* sp.	4.1 ^†^	4.9 ^†^	0.4 ^†^	1:14 ^†^	2.6 ^†^	62.8 ^†^	0.02 ^†^	0.4 ^†^	1.5 ^†^	35.3 ^†^
*Proteomonas sulcata*	1.7 ^a^	2.9 ^e^	0.2 ^d^	1:28 ^c^	0.7 ^a^	43.6 ^e^	0.31 ^b^	18.2 ^a^	0.4 ^b^	24.0 ^c^
*Rhodomonas salina*	10.4 ^e^	19.8 ^a^	1.0 ^a^	1:3 ^e^	5.5 ^d^	53.1 ^d^	1.62 ^d^	15.5 ^b^	0.3 ^b^	2.4 ^d^
*Storeatula major*	2.2 ^a^	3.5 ^d^	0.2 ^d^	1:23 ^c^	1.7 ^b^	77.3 ^b^	0.22 ^ab^	9.9 ^c^	0.03 ^d^	1.5 ^d^
*Teleaulax acuta*	0.2 ^c^	0.7 ^g^	0.02 ^e^	1:126 ^a^	0.1 ^a^	34.9 ^f^	0.01 ^c^	4.9 ^d^	0.01 ^d^	0.01 ^e^
*Teleaulax amphioxeia*	1.0 ^a^	2.3 ^f^	0.1 ^d^	1:36 ^b^	0.9 ^a^	89.6 ^a^	0.03 ^c^	3 ^e^	0.01 ^d^	0.01 ^e^

^†^ Could not be statistically tested.

**Table 5:** The sterol contents (µg sterol in mg dry weight) of the studied cryptophytes. Different letters (a–e) denote significant differences (ANOVA *p* < 0.05) between strains. The culture collection codes of the cryptophyte strains can be found in Table 1.

Species	Crinosterol µg ste in mg DW	Brassicasterol µg ste in mg DW	Stigmasterol µg ste in mg DW	Sum of Sterols µg ste in mg DW
*Chroomonas mesostigmatica*	0.93 ^a^	0.02 ^c^	0.33 ^a^	1.28 ^a^
*Guillardia theta*	nd	0.31 ^b^	0.36 ^a^	0.67 ^c^
*Hemiselmis* sp.	0.43 ^†^	1.11 ^†^	nd	1.54 ^†^
*Proteomonas sulcata*	nd	0.71 ^a^	nd	0.71 ^bc^
*Rhodomonas salina*	0.14 ^c^	0.84 ^a^	nd	0.98 ^ab^
*Storeatula major*	0.24 ^c^	0.72 ^a^	nd	0.96 ^ab^
*Teleaulax acuta*	nd	0.35 ^b^	nd	0.35 ^e^
*Teleaulax amphioxeia*	0.45 ^b^	nd	nd	0.45 ^d^

nd = not detected. ^†^ Could not be statistically tested.

## Supplementary Materials

The following are available online at www.mdpi.com/1660-3397/16/1/3/s1, Table S1: Fatty acid profiles of the studied cryptophyte strains. Concentrations are in µg FA in mg DW (± is for standard deviations).
