# Supplementary material for: Marine Cryptophytes Are Great Sources of EPA and DHA
Source: Mar Drugs. 2017 Dec 26;16(1):3. doi: 10.3390/md16010003 (PMC5793051; doi:10.3390/md16010003)
Supplement: Supplementary file 1 [file marinedrugs-16-00003-s001.pdf]

Table S1. Fatty acid profiles of the studied cryptophyte strains. Concentrations are in µg FA in mg DW (± is for standard deviations).

	Chroomonas mesostigmatica	*Guillardia theta*	*Hemiselmis* sp.*	*Proteomonas sulcata*	*Rhodomonas salina*	*Storeatula major*	*Teleaulax acuta*	*Teleaulax amphioxeia*
	CCMP/NCMA 1168	CCMP/NCMA 2712	NRC5	CCMP/NCMA 704	CCMP/NCMA 757	SM or G	SCCAP K-1486	GCEP01
14:0	0.52 ± 0.02	0.45 ± 0.06	0.57	0.66 ± 0.18	2.66 ±0.10	3.17 ± 0.09	0.25 ± 0.05	0.24 ± 0.03
i-15:0	nd	nd	nd	nd	nd	nd	0.15 ± 0.05	nd
a-15:0	nd	nd	nd	nd	nd	nd	0.01 ± 0.00	nd
i-16:0	0.09 ± 0.00	0.11 ± 0.01	0.23	0.11 ±.0.03	0.07 ± 0.00	0.09 ± 0.00	0.03 ± 0.01	0.04 ± 0.00
16:0	6.53 ± 1.23	12.8 ± 0.55	15.40	6.93 ± 0.36	3.50 ± 0.18	3 87 ± 0.15	4.04 ± 0.68	4.69 ± 0.26
16:1w9	0.01 ± 0.02	0.03 ± 0.00		0.01 ± 0.02	0.01 ± 0.00	0.01 ± 0.00	0.03 ± 0.01	0.03 ± 0.00
16:1w7	0.27 ± 0.00	0.05 ± 0.00	0.06	0.16 ± 0.02	0.17 ± 0.01	0.20 ± 0.01	0.09 ± 0.02	0.10 ± 0.01
17:0	0.10 ± 0.00	0.23 ± 0.01	0.20	0.06 ± 0.01	0.03 ± 0.00	0.02 ± 0.00	0.03 ± 0.00	0.03 ± 0.00
16:2w7	0.11 ± 0.01	0.18 ± 0.01	0.08	0.08 ± 0.02	0.15 ± 0.00	0.16 ± 0.01	0.01 ± 0.00	0.05 ± 0.00
18:0	0.37 ± 0.06	1.63 ± 0.06	1.22	0.41 ± 0.13	0.16 ± 0.01	0.14 ± 0.01	0.23 ± 0.03	0.28 ± 0.02
18:1w9	0.57 ± 0.02	0.92 ± 1.3	1.18	0.98 ± 0.23	0.07 ± 0.01	0.13 ± 0.01	0.12 ± 0.02	0.77 ± 0.17
18:1w7	1.32 ± 0.04	3.26 ± 0.28	1.84	0.50 ± 0.12	1.57 ± 0.03	1.98 ± 0.11	1.08 ± 0.18	1.70 ± 0.10
18:2w6 (LA)	2.28 ± 0.09	3.26 ± 0.31	2.59	0.75 ± 0.18	5.53 ± 0.21	1.71 ± 0.05	0.07 ± 0.01	0.92 ± 0.07
18:3w6	0.18 ± 0.00	0.24 ± 0.01	0.06	0.24 ± 0.06	3.01 ± 0.12	0.25 ± 0.01	0.13 ± 0.03	0.08 ± 0.00
18:3w3 (ALA)	13.5 ± 0.54	19.7 ± 2.12	26.20	16.4 ± 4.13	8.62 ± 0.39	13.7 ± 0.49	3.67 ± 0.67	7.03 ± 0.51
18:4w3 (SDA)	21.7 ± 0.98	19.5 ± 1.88	16.80	19.4 ± 4.96	15.4 ± 0.64	24.3 ± 0.05	11.3 ± 1.84	15.6 ± 1.12
20:0	0.08 ± 0.00	0.02 ± 0.00	0.11	0.02 ± 0.00	0.01 ± 0.00	0.01 ± 0.00	0.18 ± 0.03	0.29 ± 0.02
20:1w9	nd	nd	0.01	nd	nd	nd	nd	nd
20:4w6 (ARA)	0.06 ± 0.00	0.03 ± 0.00	0.02	0.31 ± 0.09	1.62 ± 0.09	0.22 ± 0.01	0.01 ± 0.00	0.03 ± 0.00
20:5w3 (EPA)	9.34 ± 0.48	7.14 ± 0.75	12.5	6.14 ± 1.57	5.80 ± 0.29	8.23 ± 0.32	6.58 ± 1.06	8.50 ± 0.59
22:0	0.01 ± 0.00	0.02 ± 0.00	0.02	0.01 ± 0.00	nd	nd	0.01 ± 0.00	0.01 ± 0.00
22:5w6 (DPA)	6.00 ± 0.06	2.06 ± 0.24	1.45	0.41 ± 0.10	0.25 ± 0.01	0.03 ± 0.00	nd	nd
22:5w3	0.13 ± 0.01	0.08 ± 0.01	0.31	0.18 ± 0.03	0.13 ± 0.00	0.10 ± 0.01	0.08 ± 0.02	0.06 ± 0.00
22:6w3 (DHA)	0.79 ± 0.03	1.43 ± 0.16	3.00	6.05 ± 1.41	3.77 ± 0.10	5.16 ± 0.06	3.61 ± 0.50	4.56 ± 0.25
Total sum	63.97 ± 0.99	73.06 ± 7.75	83.89	59.86 ± 13.63	52.5 ± 2.20	63.42 ± 0.76	31.75 ± 5.22	45.02 ± 3.13

* only one fatty acid sample, nd = not detected
